# Intrapopulation genetic variation in the level and rhythm of daily activity in *Drosophila immigrans*

**DOI:** 10.1002/ece3.7041

**Published:** 2020-11-28

**Authors:** Takahisa Ueno, Yuma Takahashi

**Affiliations:** ^1^ Graduate School of Science and Engineering Chiba University Chiba Japan; ^2^ Graduate School of Science Chiba University Chiba Japan

**Keywords:** daily rhythm, fruit fly, natural population, quantitative variation

## Abstract

Genetic diversity within a population, such as polymorphisms and personality, is considered to improve population performance because such intraspecific variations have the potential to alleviate the competition for a limited resource or the risk of predation and sexual harassment at a population level. Variation in the level and rhythm of daily activity in a population could also affect population performance by directly altering ecological, social, and sexual interactions among individuals. However, it remains to be elucidated whether such intra‐population variation in the level and rhythms of daily activity exists in a natural population. Here, we investigated the genetic variation in daily activity within a single natural population of *Drosophila immigrans*. We established 21 isofemale lines from a single natural population and measured larval activity level and the level and daily pattern of adult activity over a 24 hr period. Larval activity level significantly varied among isofemale lines. Likewise, the activity level in the adult stage significantly varied among lines. The significant variation was also found in the daily pattern of adult activity; some lines showed greater activity level in the daytime, and others showed greater activity level in the night. Our results consistently suggest that there is a genetic variation in behavioral activity in a natural population, probably contributing to shaping the population performance.

## Graphical abstract

Variation in the level and rhythm of daily activity in a population could also affect population performance by directly altering ecological, social, and sexual interactions among individuals. Our results suggest that there is a genetic variation in the level and rhythms of daily activity in a natural population.

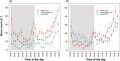

## 1 INTRODUCTION

Individuals within a population vary in many traits, including both continuous and discontinuous characteristics such as sex, color, size, morphology, behavior, and personality. Studies on intraspecific genetic variation had focused on two areas fundamental to evolutionary ecology: the evolutionary processes generating genetic variation and the ecological consequences of the evolution of genetic variation in a species/population (Bolnick et al., 2011; Forsman, 2008). For example, population genetics revealed that intraspecific genetic variation is maintained in populations through balancing selection or a migration‐selection balance (Mallet & Barton, 1989; Slatkin, 1973). Genetic variations are then suggested to enhance speciation and adaptive radiation over an evolutionary time scale (Mallet & Barton, 1989; Slatkin, 1973). On the other hand, ecological consequences of the genetic variation are getting a lot more attention (Wolf et al., 2007; Wolf & Weissing, 2012). Intraspecific (genetic and phenotypic) variation is suggested to affect ecological dynamics, such as population processes, community structure, and ecosystem function (Des Roches et al., 2018; Forsman & Wennersten, 2016). Such intraspecific variations have the potential to enhance population performance through improved use of resources (Dyer et al., 2009), parasite resistance (Sih et al., 2012), reduced predation (Ahnesjö & Forsman, 2006) and sexual harassment (Takahashi et al., 2014) at a population level. In general, variation in traits relating to predator–prey interactions or resource utilization (e.g., body colors, feeding organs, and foraging behaviors) is expected to have larger ecological effects than less functional or nonfunctional traits.

Variation in activity level and rhythms of daily activity within a population could also affect population performance by directly altering ecological, social, and sexual interactions among individuals. Variation in activity has been implicated in shaping interindividual interactions and social networks within populations (Mizumoto et al., 2017), but the ecological function of within‐population variations in daily rhythm is still controversial. In *Drosophila*, behavioral and physiological daily rhythms have been well studied under laboratory conditions (Tauber et al., 2003). Adult individuals of *D. melanogaster* have a bimodal activity distribution that is described as “crepuscular”: the flies move actively in the early morning and evening and are less active during mid‐day (Helfrich‐Förster, 2001; Tauber et al., 2003). Similar daily patterns in activity are known in other *Drosophila* species (Beauchamp et al., 2018), and such obvious crepuscular activity is thus believed to be ubiquitous in *Drosophila*. However, it remains unclear whether there are intrapopulation variations in activity patterns and their daily rhythms in *Drosophila* species. In the present study, we investigated the genetic variation in larval activity level and the level and daily pattern of adult activity within a single natural population in *Drosophila immigrans*.

## 2 MATERIALS AND METHODS

### 2.1 Study species

*Drosophila immigrans* is a globally distributed generalist which can oviposit on a wide array of substrates such as fungi, fruits, sap fluxes, and flowers (Markow & O'Grady, 2008). Their developmental time is within the range of 11–17 days, depending on temperature (Markow & O'Grady, 2006). In Japan, this species is extremely common during May and December (Beppu, 2014).

### 2.2 Fly strain

The adults of *D. immigrans* were collected in the Ecology Park of the Natural History Museum and Institute of Chiba, Japan (35°59′8″ N, 140°13′7″ E) in 2018. Each collected female was isolated to establish isofemale lines. Their siblings were maintained with media used by Fitzpatrick et al. (2007) (500 ml H_2_O, 50 g sucrose, 25 g active yeast, 8 g agar, 5.36 g KNaC_4_H_4_O_6_.4H_2_O, 0.5 g KH_2_PO_4_, 0.25 g NaCl, 0.25 g MgCl_2_, 0.25 g CaCl_2_, 0.35 g Fe_2_(SO_4_)·6.9H_2_O) in 170 ml bottles (AS‐115; Thermo Fisher Scientific). The flies were reared under a 12L:12D cycle at 23°C, at which temperature the population of *D. immigrans* is empirically known to develop well. In total, 21 isofemale lines were established in 2018. Before examining larval and adult activity, all isofemale lines were reared for three generations to reduce genetic variation within a line and to remove environmental and maternal effects. Of the 21 isofemale lines, 13 and 19 lines were used to measure the larval and adult activity, respectively (Table S1). Eleven lines were used in common for both larval and adult experiments.

### 2.3 Larval activity

Larval activity was measured between 16:00 and 18:00 during April and May in 2019. To determine the larval activity level of each isofemale line, a larva was placed on a wet filter paper (φ31 mm) without food and filmed using a digital video camera (960 × 540 pixels at 30 fps, Panasonic HC‐V480MS) for 15 min under LED light at 25°C, at which temperature we expected that larval activity is maximized (see Anreiter et al., 2016). A border around the filter paper made using a water repellent pen prevented a larva from escaping the filter paper stage. Before tracking individuals, 15‐min‐long videos were trimmed to 10‐min‐long videos. Each video was time‐compressed to a 1‐min‐long video as larval movements are very slow. Locomotor behavior was tracked using a real‐time tracking system, UMATracker. Two‐dimensional coordinate values from UMATracker were used to estimate larval activity level as the average path length of larval locomotion.

### 2.4 Adult daily rhythms

The daily activity of adult males and females of each isofemale line was examined during July 2018 and May 2019. The activity was observed for 24 hr and scored as the number of infra‐red beam breaks in 10‐min intervals using the DAM2 *Drosophila* Activity Monitor System (Trikinetics, Inc.). The activity monitor was placed in an incubator with the same settings as the rearing conditions (i.e., 12L:12D cycle at 23°C). Individual flies were anesthetized with CO_2_ and transferred into a recording tube made from a transparent straw (φ5 mm × 65 mm), one end of which was closed with rearing medium, and the other by an air‐penetrable plug. Flies were allowed to recover from the anesthetic for at least 30‐min before the recording started. We had confirmed that the LED lighting do not critically affect air temperature in an incubator.

We calculated the level of adult activity per hour by summing the number of activity counts during each hour. To resolve the problem of temporal autocorrelation in time series data, principal components analysis (PCA) was conducted for the number of activity counts in each hour. PCA condensed the 24 hr of data on adult activity into a small number of uncorrelated variables, which were the possible indices of the level and patterns of daily activity. To interpret each principal component score (PC score), isofemale lines were divided into three classes using the scores of each PC: top 4 lines, bottom 4 lines, and others.

### 2.5 Statistics

All analyses were conducted in R version 3.5.3 (R Core Team, 2019). The difference in larval activity level among isofemale lines was analyzed by one‐way ANOVA. The differences in adult PC scores among isofemale lines were analyzed by two‐way ANOVA with isofemale line ID and sex as independent variables. The *p*‐values of the two‐way ANOVA were calculated using the *F* test from the “car” package. The correlation between males and females for each PC (PC1–PC5) scores was analyzed with Pearson's correlation test, Note that, Spearman's rank correlation test was used for PC1 and PC2 scores because these males were not normally distributed (Shapiro–Wilk *W* test, PC1: *p* < 0.05; PC2: *p* < 0.01). The consistency of activity level between larval and adult stage (PC1 score) was analyzed with linear regression analysis.

## 3 RESULTS

**FIGURE 1:**
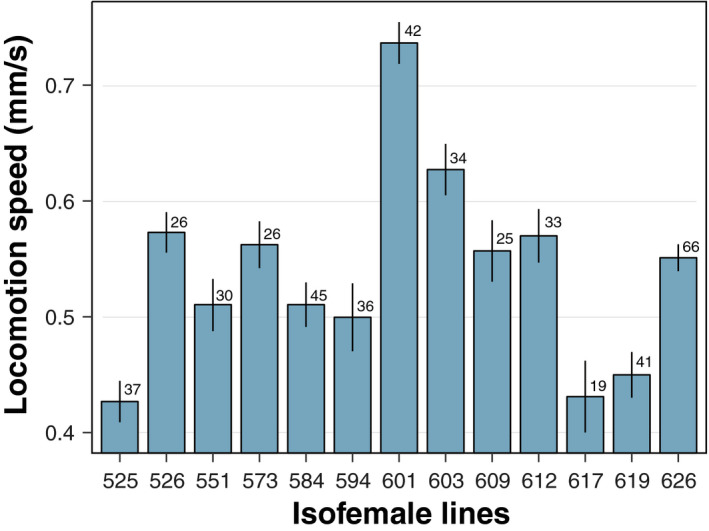
The variation in larvae locomotion speed among isofemale lines of *Drosophila immigrans*. The *x*‐axis labels are isofemale line ID, and the numeral on each bar is a sample size. Error bars are *SEM*

Larvae moved approximately 0.5 mm per second on average on the wet filter paper (Figure 1). Within‐line variation in locomotion was relatively small. The locomotion was significantly different among the 13 isofemale lines (*F*_10,387_ = 16.8, *p* < 0.001), indicating genetic variation in locomotive activity of larvae.

**FIGURE 2:**
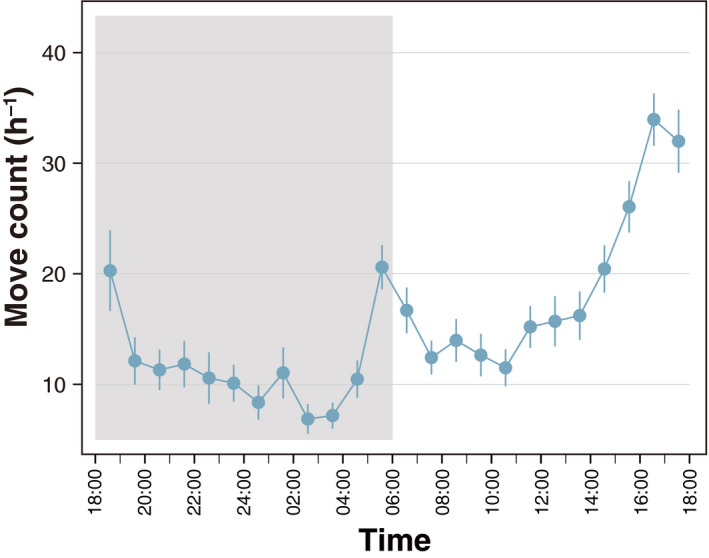
The average daily activity of all adults examined (*N* = 266). The activity of flies peaked during morning and evening. Gray area in a panel represents dark conditions. Error bars are *SEM*

**FIGURE 3:**
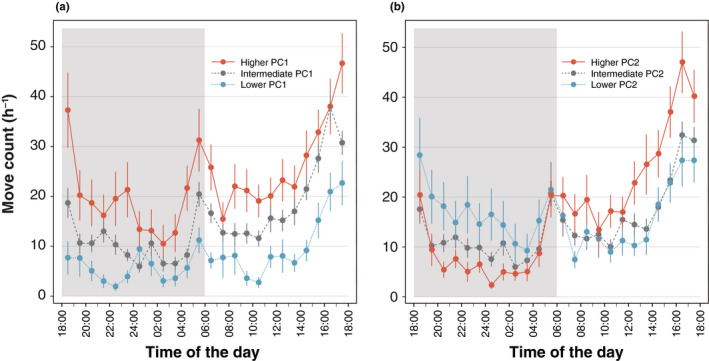
The average daily activity of adults in each isofemale line showing higher, intermediate and lower PC1 (a) and PC2 (b) values. Black and white bars represent light/ dark conditions. Solid lines (red), broken lines (green), and dotted lines (blue) represent a pattern of isofemale lines with higher PC scores, isofemale lines with intermediate PC scores and isofemale lines with lower PC scores, respectively. Error bars are *SEM*

**FIGURE 4:**
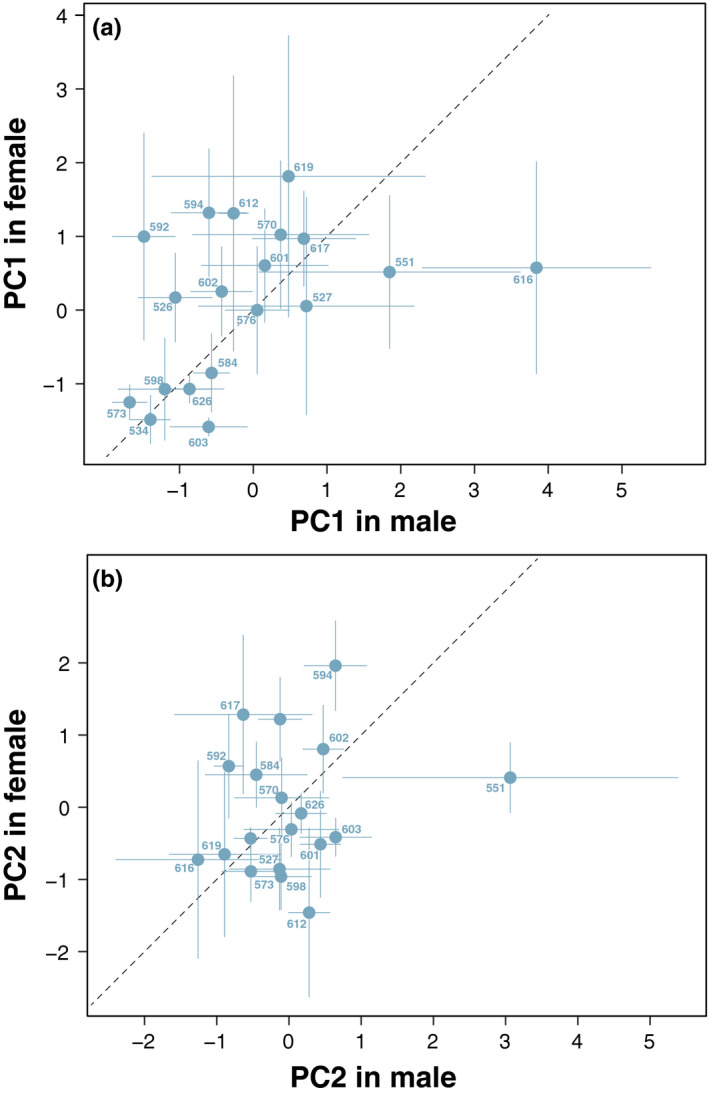
The variation in PC1 (a) and PC2 (b) and the correlations of between sexes. Dashed line represents a diagonal line. Sample sizes of male and female in each isofemale line are 6.6 ± 1.3 and 7.4 ± 1.8 (mean ± *SD*), respectively. Labels on the shoulder of each plot mean isofemale line ID. Error bars are *SEM*

For adults, the level of daily activity peaked in the early morning and evening (Figure 2). Daily activity across all measured isofemale lines across 24 hr was illustrated in Figure S1. Using 19 isofemale lines, the PCA identified two important PC axes: the first axis of the PCA explained 26.9% of the total variance (PC1) and the second axis explained 15.3% (PC2). Other PCs were less important and had a low contribution (<10%). Isofemale lines showing a high PC1 score were more active compared to those with low PC1 scores throughout a day (Figure 3a). On the other hand, lines with a higher PC2 score were more active in the light than in the dark and lines with a lower PC2 score were more active in the dark than in the light (Figure 3b). Thus, the PC1 and PC2 illustrate the overall level and daily rhythm of behavioral activity, respectively. PC1 was significantly different among the isofemale lines, while no difference was found between sexes (line: *F*_18,228_ = 2.35, *p* <0 .01; sex: *F*_1,228_ = 0.44, *p* = 0.51; interaction; *F*_18,228_ = 0.92, *p* = 0.56). PC2 also varied among the isofemale lines, but did not between sexes (line: *F*_18,228_ = 1.67, *p* < 0.05; sex: *F*_1,228_ = 0.01, *p* = 0.91; interaction; *F*_18,228_ = 1.31, *p* = 0.19). Other PCs except PC4 were also not significantly different among lines and between sexes (Table S2). Correlation between males and females was found for PC1, but not for PC2 to PC5 (PC1: *ρ* = 0.47, *p* = 0.04; PC2: *ρ* = 0.17, *p* = 0.48; Figure 4a,b; Table S3). For 11 isofemale lines of which both adult and larval activity levels were measured, the mean PC1 including both males and females was not significantly related to mean larval locomotive activity (*F*_1,9_ = 1.29, *p* = 0.28). Other mean PCs except PC4 were also not significantly related to mean larval locomotive activity (Table S4).

## 4 DISCUSSION

In *Drosophila*, daily rhythms in behavior and physiology have been well documented all over the world. Numerous studies suggest that the adults of drosophilid species show a bimodal distribution in activity (Ferguson et al., 2015; Helfrich‐Förster et al., 2020). For example, a bimodal activity pattern was reported in *D. melanogaster* (Dubowy & Sehgal, 2017; Helfrich‐Förster, 2000), *D. suzukii* (Plantamp et al., 2019), and *Zaprionus indianus* (Prabhakaran & Sheeba, 2013). Even in the present study, we showed that adult *D. immigrans* showed a bimodal distribution in activity, indicating that the daily rhythm of this species is consistent with known adult activity patterns in *Drosophila*. On the other hand, few studies had examined intrapopulation variation in adult daily activity. We here revealed the presence of intrapopulation genetic variation in the level of activity in both larvae and adults. We also found the intrapopulation genetic variation in the daily pattern of activity in the adult stage.

The activity level of each line was consistent between sexes. A previous study demonstrated that the adult activity level was positively correlated between males and females in *D. melanogaster* (Watanabe et al., 2020). The consistency of activity levels between sexes could be general in *Drosophila*. On the other hand, we showed no positive correlation between larval and adult activity of *D. immigrans*. The similar pattern has been reported in various holometabolous insects such as a red flour beetle, a mealworm, and a fruit fly; that is, no positive correlation of larval and adult activity was not found (Anderson et al., 2016; Matsumura et al., 2017; Monceau et al., 2017). A different pattern has also been reported in some hemimetabolous insects such as a damselfly and a firebug (Brodin, 2009; Gyuris et al., 2012), in which larval and adult activity tended to positively correlate each other. However, exceptionally in a behavioral polymorphism governed by *foraging* gene in *D. melanogaster*, the activity level is suggested be consistent throughout their life including both larval and adult stage (Edelsparre et al., 2014). The consistency of activity level throughout the life is still controversial in insects.

We also found that the PC2 score (the rhythm of daily activity) was not correlated between males and females, while there was significant genetic variation in the rhythm of daily activity among lines. This suggests that the daily rhythm of activity tends to differ between opposite‐sex siblings in nature. However, since few studies have addressed the variation in the rhythm of daily activity, we cannot ascertain whether such a pattern was ubiquitous at this time.

Recent studies suggest that adult *D. melanogaster* may show a different daily rhythm in semi‐natural environments. In such environments, adults dramatically increased their afternoon activity, suggesting that *Drosophila's* bimodal activity distribution may only appear under strictly controlled laboratory conditions (Green et al., 2015; Vanin et al., 2012). Thus, the variation in daily rhythms that we found under laboratory conditions may not reflect the pattern of those in a natural condition, though our findings still suggest that variation in activity rhythms themselves may exist in natural populations. The intrapopulation variation in activity rhythms may have evolved to reduce interindividual competition.

Intraspecific behavioral variation has been suggested to affect population dynamics. For example, in *D. melanogaster*, intrapopulation variation in activity improves population performance by reducing resource competition (Takahashi et al., 2018). Thus, the variation in activity that we found in the present study may be linked to the reduction of resource competition. In addition, in the present study, we found that some isofemale lines were active in the daytime and while others were active at night. The variation of activity patterns among individuals could potentially function to reduce the rate of encounters among individuals at population level. A reduction in inter‐ and intrasexual interactions, which negatively affect survival and reproduction, is likely to enhance mating success and reduce interindividual competition for resource (Hau et al., 2017; Kronfeld‐Schor & Dayan, 2003; Reebs, 2002; Závorka et al., 2016). Thus, the variation of both the level and daily pattern of activity is hypothesized to affect individual performance and thus population dynamics.

At the current moment, it is technically challenging for us to quantify the rhythm of larval daily activity. However, we have to examine the rhythm of larval daily activity to test whether its consistency with that of adult daily activity in the future. In addition, in the present study, we have not tested the effect of the presence of variation on the interindividual interactions, reproduction, and population growth. Further studies are needed to test the effect of the presence of variation in daily activity on individual performance and population process, as described above.

## CONFLICT OF INTEREST

The authors have no conflicts of interest.

## AUTHOR CONTRIBUTION

**Takahisa Ueno:** Conceptualization (equal); Data curation (lead); Formal analysis (lead); Funding acquisition (supporting); Investigation (lead); Methodology (equal); Project administration (supporting); Resources (lead); Visualization (lead); Writing‐original draft (lead); Writing‐review & editing (equal). **Yuma Takahashi:** Conceptualization (equal); Data curation (supporting); Formal analysis (supporting); Funding acquisition (lead); Investigation (supporting); Methodology (equal); Project administration (lead); Resources (supporting); Visualization (supporting); Writing‐original draft (supporting); Writing‐review & editing (equal).

## Supporting information

Appendix S1

## Data Availability

All data are available in the publicly accessible repository Dryad at https://doi.org/10.5061/dryad.h9w0vt4g1.
