# Supplementary material for: Intrapopulation genetic variation in the level and rhythm of daily activity in Drosophila immigrans
Source: Ecol Evol. 2020 Nov 28;10(24):14388–93. doi: 10.1002/ece3.7041 (PMC7771174; doi:10.1002/ece3.7041)
Supplement: Supplementary file 1 — Appendix S1 [file ECE3-10-14388-s001.docx]

**Appendix**

# Intra-population genetic variation in the level and rhythm of daily activity in *Drosophila immigrans*

Takahisa Ueno^1^ and Yuma Takahashi^2^

1. Graduate School of Science and Engineering, Chiba University, Chiba, Japan

2. Graduate School of Science, Chiba University, Chiba, Japan

**Table S1**. The list of isofemale lines of which larval and adult activity were measured.

| Line ID | Larval activity | Adult activity |
| --- | --- | --- |
| 525 | Measured | Not measured |
| 526 | Measured | Measured |
| 527 | Not measured | Measured |
| 534 | Not measured | Measured |
| 551 | Measured | Measured |
| 570 | Not measured | Measured |
| 573 | Measured | Measured |
| 576 | Not measured | Measured |
| 584 | Measured | Measured |
| 592 | Not measured | Measured |
| 594 | Measured | Measured |
| 598 | Not measured | Measured |
| 601 | Measured | Measured |
| 602 | Not measured | Measured |
| 603 | Measured | Measured |
| 609 | Measured | Not measured |
| 612 | Measured | Measured |
| 616 | Not measured | Measured |
| 617 | Measured | Measured |
| 619 | Measured | Measured |
| 626 | Measured | Measured |

**Figure S1.** The daily activity of adults of each isofemale line. The activities of flies were varied among lines. Gray area in a panel represents dark conditions. Error bars are SEM.

**Table S2**. Results of two-way ANOVA for PC3 to PC5. The values in parentheses are the contribution (proportion of variance) of each PC score.

| PC3 (6.2%) |  |  |
| --- | --- | --- |
|  | ***F*** | ***p*** |
| line | 1.10 | 0.35 |
| sex | 3.2 | 0.07 |
| line × sex | 0.57 | 0.92 |
|  |  |  |
| **PC4 (5.3%)** |  |  |
|  | ***F*** | ***p*** |
| line | 1.68 | 0.04 |
| sex | 0.72 | 0.40 |
| line × sex | 1.14 | 0.31 |
|  |  |  |
| **PC5 (4.7%)** |  |  |
|  | ***F*** | ***p*** |
| line | 1.48 | 0.10 |
| sex | 12.1 | <0.001 |
| line × sex | 1.43 | 0.12 |

**Table S3**. Correlation tests between males and females for PC3, PC4, or PC5 scores analyzed by Pearson’s correlation.

|  | ***r^2^*** | ***p*** |
| --- | --- | --- |
| **PC3** | 0.12 | 0.15 |
| **PC4** | 0.03 | 0.48 |
| **PC5** | 0.0007 | 0.91 |

**Table S4**. Simple linear regression analyses between larval activity and each PC scores (PC2–PC5).

| **independent variable** | **dependent variable** | ***F*** | ***p*** |
| --- | --- | --- | --- |
| **larvae activity** | **PC2** | 0.19 | 0.67 |
|  | **PC3** | 0.17 | 0.69 |
|  | **PC4** | 7.03 | 0.026 |
|  | **PC5** | 0.04 | 0.84 |
